# Establishment of a self-propagating population of the African malaria vector *Anopheles arabiensis *under semi-field conditions

**DOI:** 10.1186/1475-2875-9-356

**Published:** 2010-12-08

**Authors:** Kija RN Ng'habi, Dickson Mwasheshi, Bart GJ Knols, Heather M Ferguson

**Affiliations:** 1 Biomedical and Environmental Thematic Group, Ifakara Health Institute, Box 53, Ifakara, Tanzania; 2 Wageningen University and Research Centre, Wageningen, The Netherlands; 3 Division of Infectious Diseases, Tropical Medicine & AIDS, Academic Medical Center, F4-217, Meibergdreef 9, 1105 AZ Amsterdam, The Netherlands; 4 K&S Consulting, Kalkestraat 20 6669 CP Dodewaard, The Netherlands; 5 Institute of Biodiversity, Animal Health and Comparative Medicine, University of Glasgow, G12 8QQ, Glasgow, UK

## Abstract

**Background:**

The successful control of insect disease vectors relies on a thorough understanding of their ecology and behaviour. However, knowledge of the ecology of many human disease vectors lags behind that of agricultural pests. This is partially due to the paucity of experimental tools for investigating their ecology under natural conditions without risk of exposure to disease. Assessment of vector life-history and demographic traits under natural conditions has also been hindered by the inherent difficulty of sampling these seasonally and temporally varying populations with the limited range of currently available tools. Consequently much of our knowledge of vector biology comes from studies of laboratory colonies, which may not accurately represent the genetic and behavioural diversity of natural populations. Contained semi-field systems (SFS) have been proposed as more appropriate tools for the study of vector ecology. SFS are relatively large, netting-enclosed, mesocosms in which vectors can fly freely, feed on natural plant and vertebrate host sources, and access realistic resting and oviposition sites.

**Methods:**

A self-replicating population of the malaria vector *Anopheles arabiensis *was established within a large field cage (21 × 9.1 × 7.1 m) at the Ifakara Health Institute, Tanzania that mimics the natural habitat features of the rural village environments where these vectors naturally occur. Offspring from wild females were used to establish this population whose life-history, behaviour and demography under semi-field conditions was monitored over 24 generations.

**Results:**

This study reports the first successful establishment and maintenance of an African malaria vector population under SFS conditions for multiple generations (> 24). The host-seeking behaviour, time from blood feeding to oviposition, larval development, adult resting and swarming behaviour exhibited by *An. arabiensis *under SFS conditions were similar to those seen in nature.

**Conclusions:**

This study presents proof-of-principle that populations of important African malaria vectors can be established within environmentally realistic, contained semi-field settings. Such SFS will be valuable tools for the experimental study of vector ecology and assessment of their short-term ecological and longer-term evolutionary responses to existing and new vector control interventions.

## Background

In Africa, current frontline strategies for reducing malaria transmission rely on the use of residual insecticides through application on insecticide-treated nets (ITNs) and indoor residual spraying (IRS). The contribution of these strategies to reduce child mortality and morbidity has been considerable [1-4]. However, these approaches are facing challenges and limitations as the mosquito vectors they target are increasingly becoming resistant to insecticides [5,6] and many exhibit behavioural plasticity (e.g. biting and resting outside of houses, or early in the evening) that limits their contact with insecticides [7], indicating that these strategies alone may not be sufficient and that new control strategies are needed to supplement them [8].

One of the challenges undermining contemporary vector control strategies is the limited understanding of the ecological complexities that allow vector populations to persist and evade control approaches. Taking the example of African malaria vectors, there is insufficient understanding of mosquito life-history processes that occur outside of the domestic environment (e.g. houses) where they usually bite, including oviposition, larval development, sugar feeding, mating and dispersal [8-10]. Most vector control studies are understandably focused on developing and evaluating specific interventions. While such studies provide the ultimate evidence for evaluating whether to adopt a particular strategy, failure to concurrently measure the ecological parameters of the target vector population during the trial means that little evidence is available to interpret why an intervention failed, and what aspects of its implementation could be modified to achieve greater success. Paying explicit attention to mosquito ecology is vital not only for interpretation of why some otherwise well proven interventions are less effective than expected in different ecological settings, but also for identifying other vulnerabilities in the mosquito life cycle that could be targeted by novel methods.

Gaining insight into the ecological processes of malaria vectors can be both logistically difficult and expensive in natural field settings. This is due to the lack of sampling tools for reliably measuring the abundance and behavioural diversity of different species, genotypes, sexes and life-history stages of mosquito vectors inside and outside of domestic environments, and because of the substantial heterogeneity in their density over time and space [11,12]. As a result of these inherent challenges, many researchers adopt a laboratory experimental approach to quantify key aspects of mosquito life-history and demography. However, it is recognized that laboratory conditions may be insufficient to adequately represent vector fitness and behaviour in nature. Furthermore, the artificial feeding and rearing regimes used in laboratory colonies have been associated with the appearance of behaviours [13,14] and phenotypic traits [15] that are atypical of corresponding field populations. Consequently, in order to progress understanding of vector ecology beyond the limitations of current field and laboratory approaches, there is an urgent need for more environmentally realistic experimental systems where mosquito vector behaviour, ecology and population dynamics can be studied in a natural context over multiple generations.

Contained semi-field systems (SFS) have been proposed as more realistic and reliable experimental tools for the characterization and manipulation of vector ecology [16,17]. An SFS is defined as an experimental mesocosm, situated within the natural environment of the target vector population and exposed to similar climatic conditions, within which all natural dietary and habitat resources for their life-cycle completion are present [16,17]. The movement of insect vectors into or out of the SFS is typically prevented by netting which blocks their dispersal, but not natural airflow or climatic influences. A key benefit of SFS is that they permit the maintenance of relatively large vector populations in a situation where mating and other behavioural activities can occur more naturally than in the laboratory, and where inbreeding may be less likely to occur. It is thus expected that the demographics, genetic composition, behaviour and life-history of vectors maintained under SFS conditions will be much more representative of wild populations than laboratory colonies. Another advantage of such systems is that in contrast to field studies, the exposure of workers to pathogens such as malaria can be eliminated (e.g by restricting access to potential sources of infection). In the absence of such risk, researchers can conduct a wider range of experimental manipulations, including exposure to mosquito biting, that would be ethically unacceptable in the field. Finally by facilitating detailed study of a defined vector population over time, SFS provide a unique opportunity to investigate their evolutionary as well as ecological dynamics in response to experimental manipulations that mimic the effect of predicted environmental change or interventions; a feat very difficult to achieve under natural field conditions.

Although the value of SFS as an experimental tool for insect disease vectors is increasingly recognized [17-21], few such systems have been successfully established [16,17], and none so far have reported the successful maintenance of a vector population for multiple generations. Here we report the first successful long-term establishment (> 24 generations) of an African malaria vector population under SFS conditions within an area of endemic transmission in southern Tanzania [17]. The study focused on the establishment of *An. arabiensis*, a widespread vector of malaria in Africa [22,23]. Historically, *Anopheles gambiae s.s*, has been recognized as the most important vector of malaria in Africa. However, the abundance and distribution of this vector species is shrinking in many parts of the continent following the widespread use of ITNs, with its sister species *An. arabiensis *playing an increasingly important role in maintaining transmission [23-25]. This is because *An. gambiae s.s*. is more endophilic while *An. arabiensis *is more exophilic and less susceptible to indoor control measures. Given the growing importance of *An. arabiensis*, there is increased interest in obtaining knowledge of its ecology to stimulate new approaches for its control.

## Methods

### Experimental set-up

A large netting-enclosed semi field system (SFS) was constructed for the study of malaria vector ecology at the Ifakara Health Institute (IHI) in southern Tanzania [17]. This 1800 m^3 ^facility is situated on the main campus of the IHI which is within the Kilombero valley (7'44"- 9'29"S/35'33" -36'56" E), an area of high malaria endemicity where intense levels of transmission are maintained year round [26]. Although all of the three main major African vector species are found in this area (*Anopheles arabiensis, An. funestus *and *An. gambiae s.s*.), transmission is largely dominated by *An. arabiensis *which composes >85% of the vector population in most areas [25].

**Figure 1:**
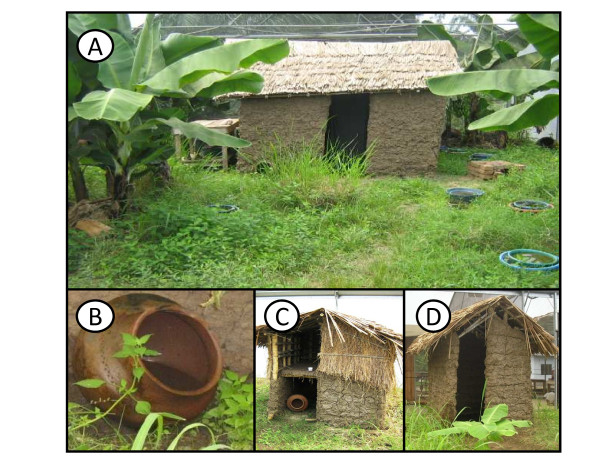
**The inside of the SFS experimental chamber where the *An. arabiensis *population was established, showing:** (**A**) mud-walled house, natural vegetation and planted food crops, (**B**) clay pots used as outdoor resting sites, (**C**) chicken coop and (**D**) cow shed.

One large experimental chamber (21 × 9.1 × 7.1 m) of the SFS was designated for the establishment of a long-term *An. arabiensis *population. This chamber is enclosed from the surrounding environment by PVC-coated polyester netting (346 holes per inch^2^, Polytex UK) with the interior set up to mimic the natural habitat features of the rural village environments where *An. arabiensis *are typically found. The floor of the chamber was covered with 30 cm of soil obtained from the nearby area, and the vegetation emerging from seeds therein was allowed to grow naturally (Figure 1a). In addition, a variety of other food crops which are normally cultivated around rural homesteads were also planted in the system (e.g. banana plants [*Musa paradisiacal*], papaya [*Carica papaya*], and sweet potatoes [*Ipomoea batatas*]). Furthermore, castorbean plants (*Ricinus communis*), on which anopheline mosquitoes have been observed to rest and feed upon in East Africa [27,28] were also planted. Although the outer walls of the experimental chamber were separated from the surrounding environment by netting, the decision was taken to cover the roof with polyethylene plastic (plastic film, Filclair Serren Industry N.V.) to provide flexibility to experimentally manipulate rainfall in future research, and also to protect the chamber from the rare but extremely heavy rains that occasionally occur in Kilombero. Consequently, vegetation within the system was watered by sprinklers three times each week. A traditional mud walled house, cow shed and a chicken coop (Figure 1a, c, d) were constructed following local design to provide adult resting sites [17]. Clay pots (n = 23) partially filled with water (to provide humidity) were also distributed throughout the chamber (Figure 1b) to provide additional refuge sites to adult mosquitoes [29]. Clay pots were made locally following the design typically used for water storage and cooking in Kilombero.

**Figure 2:**
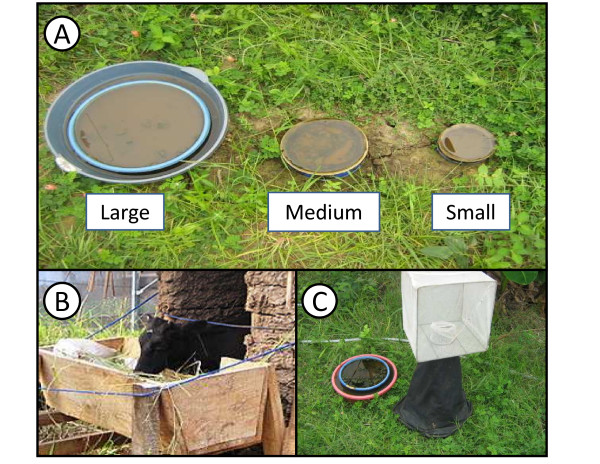
**Mosquito habitat features within the SFS: (A) Larval habitats of large (diameter 43.5 cm, maximum depth 5 cm), medium (diameter 19.5, maximum depth 4 cm) and small size (diameter 13.5, maximum depth 3 cm) within the SFS, (B) a calf host used to provide blood meals to adult females, and (C) a trap for collecting adults emerging from a larval habitat**.

In the wild, *An. arabiensis *typically lay their eggs in small, shallow sunlit water pools ranging in size from puddles up to large swamps [30,31], and can include man-made as well as natural water holding bodies that are free from canopy cover [32,33]. To mimic natural sites, artificial larval habitats (Figure 2a) of variable sizes (large, medium and small) were made by half filling plastic basins with a base of soil, and then adding water to surface level (Figure 2a). This design gave females the opportunity to land and lay their eggs on wet soil or shallow water as they do in nature [34]. The soil layer acted as a source for microbial and/or algal growth which provides food for larval growth and development [35]. Twenty large artificial larval habitats (diameter 43.5 cm, maximum depth 5 cm) were made by burying plastic containers to ground level. In addition, five medium-sized (diameter 19.5, maximum depth 4 cm) and five small (diameter 13.5, maximum depth 3 cm) artificial larval habitats (Figure 2a) were also distributed throughout the compartment. Water depth was maintained in these habitats through daily replenishment with tap water.

### Establishment of *An. arabiensis*

The population of *An. arabiensis *established in the SFS was founded from a wild population in the nearby village of Sagamaganga (~20 km from the IHI, -8.0667 S; 36.8000 E). This village is situated along the flood plains of the Kilombero River where anopheline larval habitats are abundant. Pilot work had shown that *An. arabiensis *constitutes approximately 90% of the *An. gambiae s.l*. complex in this area (Mayagaya & Ferguson, pers. comm). Live blood-fed females that were morphologically identified as *An. gambiae s.l*. were collected from houses and animal sheds using mouth aspirators in May 2008. Collections were made continuously until enough females were obtained to produce the target number of larvae for release into the SFS (provisionally set at 3000 for release over one week in May 2008). On the day of their capture, blood-fed females were transported to the IHI semi-field insectary where they were transferred into individual cups for oviposition. After oviposition, wild-collected females were killed and subjected to PCR analysis to confirm their species [36]. Larval offspring of all females (N = 560) identified as *An. arabiensis *were pooled and continuously added to the artificial larval habitats in the SFS during the week of release (Figure 1c).

Prior to the release of larvae into the SFS, a variety of other invertebrates that naturally occur in and around houses in rural Tanzania (e.g. praying mantids [Mantidae], grasshoppers [Acridoidea], ants [Formicidae]) and important *Anopheles *predators such as jumping spiders (Salticidae) [37] were observed in the SFS. These predators probably entered the system in soil and building material during the chamber set-up, and were allowed to establish within it. After introduction, larvae were not provided with any source of food other than the micro-organisms growing naturally within larval habitats.

### Adult maintenance and blood feeding

Adult mosquitoes emerging from larval habitats were allowed to fly freely in the SFS and feed on plant and vertebrate host sources available within it. A major issue when establishing long-term mosquito populations in SFS is to ensure containment and prevent the accidental introduction of malaria parasites that could infect mosquitoes and pose an infection risk to researchers. To achieve this, four steps were routinely undertaken. First, mosquito containment was ensured by the installment of a triple door entry-system which prevented direct entrance or accidental mosquito escape (each door is opened and closed independently). Second, the integrity of the outer netted walls and roof was checked thrice weekly during routine inspection. Third, blood meals were provided only from calves (which *An. arabiensis *feed on as well as humans under natural conditions; Figure 2b). As cattle are dead-end hosts for human malaria parasites, mosquitoes that have fed only on them are incapable of becoming infected or transmitting parasites. Host blood was provided to mosquitoes by introducing a calf into the SFS every evening from 7.00 PM - 7.00 AM for five consecutive nights each week. Finally, all research staff working in the area were screened for malaria parasites on a weekly basis using a rapid diagnostic kit before being allowed to enter the SFS. Any staff that tested positive for malaria were immediately given a full course of anti-malarial treatment (artemisinin combination therapy), and restricted from entering the SFS for at least 2 weeks after their infection was cleared.

### Regular entomological monitoring

Daily temperatures inside microhabitats within the SFS was monitored by placing data loggers (Tiny tag™) in aquatic larval habitats and potential adult resting sites (the house, cow shed, chicken coop and clay pots). Water temperature was monitored only in large and small larval habitats by submerging data loggers within them (Tiny tag™). After the introduction of *An. arabiensis*, larval habitats were inspected daily for the presence of larvae and pupae. Larval development was monitored for eighteen days from the day that first instars were released into ten larval habitats. Emerging adults were monitored by setting up emergence traps (Figure 2c) over all large larval habitats. Emerging adults captured in these traps were counted and then released into the SFS. *Anopheles arabiensis *population growth over the first five generations within the SFS was assessed from the number of adults emerging (measured by capture from emergence traps). The length of time between consecutive mosquito generations was also estimated. This was done by adding together the number of days from the time of blood-feeding to the first observation of first instars within larval habitats, the subsequent number of days required for these first instars to emerge as pupae, and the estimated number of days between pupal emergence and the resultant females taking their first blood meal.

An experiment was set up to assess the effect of larval habitat size on larval survival. Here, subsets of large, medium and small larval habitats were covered with netting to prevent oviposition from free flying females. A fixed number of 100 first instar larvae (from gravid females collected in the SFS) were added to these habitats to monitor their development. This experiment was run from the 5^th^-14^th ^generation to achieve a total of twenty replicates from each larval habitat size class. Habitats to which larvae were added were checked on a daily basis, with the total number of pupae that successfully developed being counted. These pupae were subsequently transferred to another larval habitat in the SFS for emergence.

The resting behaviour of both males and females was assessed by counting the number of adults observed inside the mud house, cow shed and chicken coop (all considered 'indoor' habitats), and the clay pots (outdoors) during the day with the aid of a flash light and counter. The assessment was conducted for three consecutive days from generation 2 to 7, and was repeated again at generation 24. The age structure of males and females in the SFS was estimated from a random sub-sample of 35 males and 176 females that were collected at generation 20. Dissections were performed on this subset to assess their approximate age from their reproductive morpohology, with female age being estimated from parity status as indicated from their ovaries [38] and male age from the number of spermatocysts and morphology of their accessory glands [39]. Swarming behaviour was also assessed by daily inspection for aggregations of males in flight at dusk (between 7-8 pm).

### Data analysis

Analysis of variance (ANOVA) was used to assess temperature differences between larval habitats and resting sites inside the SFS using the SPSS statistical package (13.0 for windows). Generalized linear models were used to test whether larval survival (as assessed by the proportion of pupae emerging from the 100 instars initially present) varied between larval habitats of different size classes (R statistical software). Here 'habitat size' was treated as a main effect, and the generation on which observations were made as a random effect. The daily larval survival rate in large larval habitats was also estimated using the formula S = *P*^1/t^, where *p *is the proportion of first instar larvae that survive to pupation and *t *is the mean time to pupation in days [40]. Similarly, generalized linear models were used to test whether the proportion of *An. arabiensis *adults resting in indoor versus outside habitats varied between sexes.

## Results

### Microclimatic conditions

Average water temperatures within the large and small larval habitats were within 1.2°C of one another (Table 1). Due to the limited availability of data loggers, it was not possible to simultaneously measure the temperature inside all of the four potential adult resting sites and outside air conditions in the SFS, however measurements made within these microhabitats at different times of year suggested that all fell within the natural temperature range of *An. arabiensis *(Table 1). Concurrent measurements made within the mud house, cow shed and chicken coop over a one week period in July 2010 indicated that air temperature varied significantly between these resting sites (*F*_2, 2263 _= 45.2, *P *< 0.001, Table 1). Temperature within the chicken coop was approximately 1°C warmer than the mud house (*P *< 0.001) and cow shed (*P *< 0.001) respectively, with no significant difference in temperature between the house and cow shed (*P *= 0.71).

**Table 1:** Mean temperatures (°C) of mosquito larval habitats and adult resting sites inside the SFS.

Description	Mean temperature (°C)	Period of measurement
*Larval habitat*		
Large size	24.9 (0.05)	July 1^st ^- Oct 15 2008
Small size	25.3 (0.03)	Oct 15-21 2008

*Resting site*		
Inside mud house	24.39 (0.17)	16-28 July 2010
Inside cow shed	24.23 (0.09)	16-28 July 2010
Inside chicken coop	25.43 (0.09)	16-28 July 2010
Outdoor clay pot	25.47 (0.06)	15-30 Oct 2008
Air temperature	31.30 (0.28)	Feb 29-Mar 9 2008

Values in brackets represent one standard error.

### Larval development and population growth

**Figure 3:**
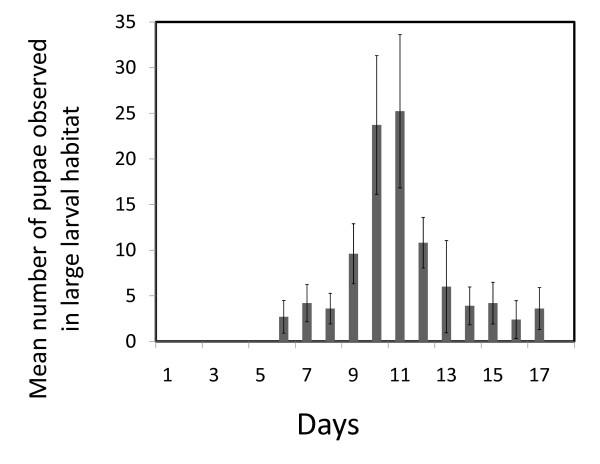
**The mean daily number of pupae observed in a subset of the large larval habitats (n = 10) in the SFS starting from the first day that 1^st ^instar larvae were released**. Bars represent one standard error.

The time required for larvae to develop and pupate after the first release into large habitats ranged from 6 to 17 days, with a median time of 11.5 days (Figure 3). Although the exact number of generations that passed since the founder generation could not be precisely established due to overlapping generations, we conservatively estimated the average time between successive generations as 22 days. This was computed by summing the estimated median development time from 1^st ^instar larvae to pupae (11.5 days), the assumed time from pupation to adult emergence (2 days [41]), the estimated time from adult emergence to blood feeding (estimated at an average of 3 days under field conditions [42]), and the estimated number of days it took from the time that females blood fed in the SFS to the first appearance of 1^st ^instar in larval habitat (5 days).

**Figure 4:**
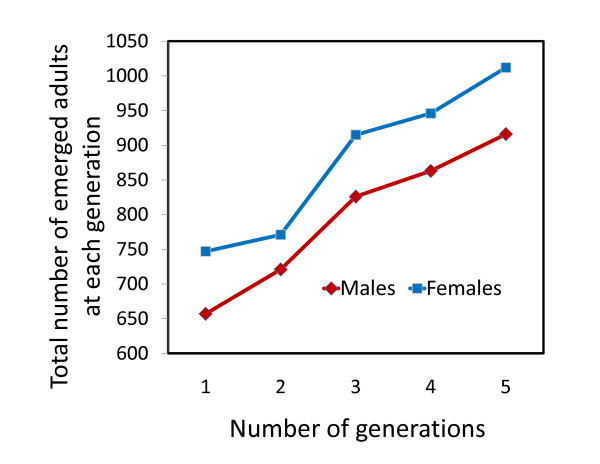
**The cumulative number of adult mosquitoes collected in emergence traps from large larval habitats (n = 20) in the SFS over the first 5 consecutive generations of this study**.

**Figure 5:**
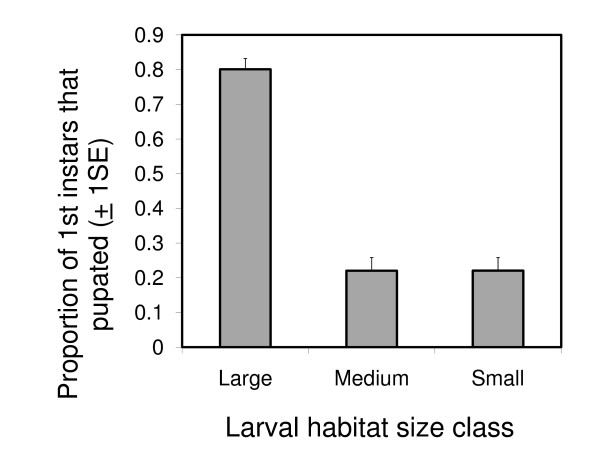
**The proportion of 1^st ^instar larvae that survived to pupate in large, medium and small larval habitats within the SFS (bars indicate one standard error)**.

The number of pupae emerging within the SFS was observed to increase during the first five generations, indicating that the SFS population was growing (Figure 4). The survival of larvae to pupation varied significantly between different larval habitat size classes (χ_2_^2^= 62.44, p < 0.001 Figure 5). Specifically, the pupation rate in the large-sized habitats (69%) was almost 4 times greater than in the small and medium habitats (Figure 5). The daily larval survival rate in large larval habitats was estimated to be 0.962 per day. Due to the labour intensive nature of performing daily larval abundance and adult resting behaviour surveys, the population size in the SFS was monitored for only 5 out of the 24 generations monitored here. However, a general approximation of the average abundance of each *An. arabiensis *generation could in principle be obtained by summing up the number of emerging adults daily in all larval habitats, collected in emergence traps over each 22 day period (assumed generation length).

Larval predation by ants was observed in the SFS. On several occasions ants were observed on the sides of aquatic habitats, carrying parts of mosquito larvae or adults that had probably been attacked during eclosion. Initially, an attempt was made to reduce ant-related predation by placing larval habitat bowls within a second water-filled outer bowl to provide a protective moat (Figure 1c). At first larvae were only observed within the inner bowl of larval habitats. However from the 3^rd ^generation onwards, females began ovipositing within moat barriers and also inside the shallow water pools within clay pots (Figure 1b), thus ant-predation could not be completely prevented.

### Adult feeding and survival

Of the 35 dissected males which were collected on the same day from the SFS (generation 20), only four males (11.4%) were classified as being in the ≤ 4 days old age category [39]. The remaining 31 males (88.8%) had a number of spermatocysts ranging from 0-2 which are estimated to correspond to an age of > 4 days post-emergence. Of the 176 females collected, 42 and 26 were observed to be either blood fed or gravid respectively and were not dissected. The ovaries of the remaining 108 unfed females were dissected to determine their reproductive history. Within this sample, 38 (35.2%) were virgins, 40 (37.04%) nulliparous and 30 (27.8%) had previously laid eggs. It was not possible to precisely age-grade the parous class into gonotrophic cycles.

### Resting behaviour and reproduction

**Figure 6:**
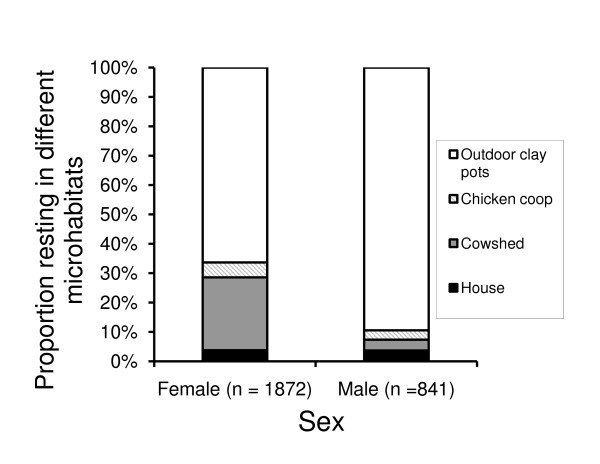
**The proportion of male (n = 841) and female (n = 1872) *An. arabiensis *adults found resting in different sites within the SFS**.

Both sexes (male [n = 841] and female [n = 1872]) of *An. arabiensis *adults were observed in all resting sites (Figure 6). While both sexes were more frequently found resting in the outdoor clay pots than in indoor environments, males were substantially more exophilic than females (χ_3_^2 ^= 223.05, P < 0.001, Figure 6). Restricting analysis to indoor resting mosquitoes, females were more likely to be found resting in the cow shed than in the house or chicken coop (χ_2_^2 ^= 433.93, p < 0.001). Unlike females, males were less selective in their use of indoor resting sites (χ_2_^2 ^= 0.39, p = 0.83, Figure 6). During generations 4 and 5, observations were made 3 times each week for the presence of adult mating swarms (at dusk). Of the 24 nights on which observations were made, swarms were detected on 14 occasions (58.3% of the time). When observed, swarms consisted of 50-100 males that formed at dusk (at approximately 7.30 PM, on one half of the SFS where the horizon was visible), and lasted for 15-20 minutes. Also males and females in copula were observed in these swarms.

## Discussion

This study reports the first successful establishment of a self-replicating population of an African malaria vector, *An. arabiensis*, in a contained semi-field system. It is suggested that a major contributing factor to the successful establishment of this population was the close concurrence between the environmental conditions of the SFS and those experienced by mosquitoes in nature. The mean daily temperatures recorded in all SFS larval habitats were within the range reported for natural *An. gambiae s.l*. larval habitats (20°C-36°C) [43,44] and never exceeded their upper tolerance limit of 40°C [34,44,45]. The time required for *An. arabiensis *to develop from larvae to pupae within these habitats (6 - 17 days) was similar to the reported range (8-18 days) for *An. gambiae s.l*. in the wild [46]; with the median larval development under both our SFS (11.5 days) and field conditions (e.g. 11.9 days [41]) being close. During their development, the only source of food that would have been available to larvae was microbes and algae that developed naturally within their aquatic habitats. These resources are the primary food source of larvae in natural populations [35], and likely played a similar role in the SFS.

The survival of larvae to pupation within the SFS was highly dependent on larval habitat size, with the pupation rate in 'large' habitats being four times higher than in medium and small habitats. The pupation rate within large habitats corresponds to a daily larval survival of 96.2%, which is in line with what has been reported in other semi-field settings (95.7%) [40]), but slightly higher than what has been reported in some field populations (e.g. 85% [47]). The moderately higher pupation rate observed here may be a result of reduced predation and competition within our SFS relative to field conditions. It is hypothesized that the enhanced pupal productivity of the 'large' larval habitats in the SFS is a function of the greater amount of food resources (algae and microbes) they can support relative to smaller habitats. In the absence of predators and pathogens, food availability within larval habitats is a key predictor of the number of adults that emerge from them [43]. The total quantity of microbial growth in larval habitats is related to both surface area and volume [30] and thus are greater in the larger than small habitats where larvae may have experienced more intense resource competition.

Similar to larval development, the behaviour of adult mosquitoes within the SFS was also consistent with what has been reported in nature. The mean temperatures within available adult resting sites varied by no more than 1.3 °C, and all were within the tolerance range of *An. arabiensis *[48,49]. However, there was considerable variation in resting preference. Both males and females were more likely to rest in clay pots (outdoors) than in indoor sites; confirming the previously demonstrated exophilic tendency of this species in the wild [50,51]. Although *An. arabiensis *is known to be substantially more exophilic than *An. gambiae s.s*., it has not previously been possible to estimate the relative proportion of outdoor resting within natural populations. If our SFS results are typical of natural populations, it suggests that up to 60% of resting adults may be missed by surveys and control measures targeted indoors. Restricting consideration to indoor resting sites, females were more selective than males. Specifically, females were more likely to rest in the cow shed than in other sites, whereas indoor resting males occurred with similar frequency in all three resting sites. The closer association of females with the cow shed suggests they prefer to rest close to the host blood source, and may be most efficiently targeted there.

Several other aspects of the adult behaviour and life history of *An. arabiensis *within the SFS conformed to what is known of their natural ecology. For example, female *An. arabiensis *in the SFS readily blood-fed on cows and were able to maintain their population exclusively on this host type as has been reported in other field populations in East Africa [52]. In nature, plants are thought to be a major source of sugar for both male and female mosquitoes [27,28]. Mosquitoes can readily imbibe and digest plant juices and nectar to enhance their survival [53,54]. As males rarely survive for more than 48 h without a sugar source [54], evidence that a large proportion of adult males (at generation 20) within the SFS (80%) were four days or older indicates that they were feeding on plant nectar sources within it. These plant sugar sources may also have been used as nutritional supplements by females. Further studies such as gut content analysis could confirm the extent of *An. arabiensis *reliance on plant nectar, and which types are preferred.

Analysis of the age structure of male and female *An. arabiensis *at generation 20 indicated that a significant proportion of both male and females survived beyond the minimum period required to reproduce. In this study, approximately 80% of males were estimated as being > 4 days, the period beyond the peak of *An. gambiae s.l*. mating activity [55,56]. Similar analysis of adult females indicated that 28% of females had survived through their first gonotrophic cycle (estimated as < 4 days). The observed parous rate of 0.28 corresponds to an estimated daily survival rate of 0.53, which falls within the range of reported for adult *An. gambiae s.l*. daily survival during the dry (0.49) and wet seasons (0.84) in East African populations [25,57,58]. Although the age-grading methods used in this study provide a general indication of adult mosquito age, they could not precisely estimate how long individuals survived beyond four days. Further age-grading studies using chronological age estimation methods to more precisely estimate the life span are recommended [59].

Another natural adult behaviour observed within this SFS population was swarming. Swarming has been suggested as the primary reproductive strategy of *An. gambiae s.l*. mosquitoes [60,61], and has been documented in several wild populations in East [62] and West Africa [61,63]. However in some parts of East Africa, male anopheline swarms have been difficult to observe, possibly due to the fact that they occur at dusk when visibility is poor [61,62,64], or because these populations deploy alternative strategies such as mating indoors [65]. Due to its inconsistent occurrence (observed on 58.3% of occasions), we could not establish whether swarming was the primary mating strategy of mosquitoes in the SFS. Further investigation is needed to identify the mating strategies of *An. arabiensis *both in the SFS and the wild population from which they were established.

The SFS approach adopted provides useful opportunities for characterizing mosquito demography, life-history and behaviour traits that are difficult to measure in nature, and poorly represented in the laboratory [11,66]. This advantage will be particularly strong for vector species that are difficult to colonize under laboratory conditions (e.g. *An. funestus *and *Mansonia annulata*) and/or sample in the wild; as the relatively more natural conditions within the SFS may prove more amenable for their establishment. Although it is argued that biological inferences generated in SFS provide a more accurate representation of field populations than laboratory colonies, this approach also has limitations. For example in the SFS, there were no interactions between *An. arabiensis *and other mosquito species in larval habitats as occurs in nature [67]. Furthermore the high availability of aquatic sites within the SFS probably minimized cannibalism and other types of intraspecific competition [68]. Also unlike field settings, pressure from insecticides or other vector control interventions was absent in the SFS, and the diversity of natural predators and competitors was probably under represented. Furthermore although humans are a common host for *An. arabiensis *in many African settings, this host type was not available in the SFS during their typical host-seeking period (e.g. 10 pm -5 am [69]). Consequently, *An. arabiensis *within this system were not exposed to human malaria parasites, which may have eliminated another source of selection pressure that acts on natural mosquito populations. However, as only 1-2% of *An. gambiae s.l*. become infected even in highly endemic settings, it is perhaps unlikely that parasites are a significant source of selection [70]. Finally, there have been some accounts that the host preference of vectors (from an insectary population) when assayed under semi-field conditions give a biased representation of natural feeding preferences [14]. Whether SFS populations that have been established directly from a wild population and consistently maintained on natural host types may also develop atypical preferences is not yet unknown, but careful and repeated monitoring is required to ensure that these and other behavioural traits of SFS populations reliably approximate natural vector ecology. Supplementing stable population within SFS routinely with fresh materials from the field may be another option to circumvent this, but requires further study. Thus it is cautioned that SFS studies should be seen as a bridging ground between lab and field, and not replacement of field studies. Furthermore, where possible observations from these SFS studies should be verified under natural conditions.

In addition to elucidating fundamental aspects of vector ecology, SFS studies have a variety of other potential uses including the preliminary evaluation and optimization range of vector control interventions and trapping methods. SFS can be used to develop and optimize new field sampling tools and trapping methods, and identify the most promising candidates to take forward for full field testing within a relatively short period of time (e.g. optimal doses for repellants/attractants [71]). Additionally, the long-term establishment of vector populations allows the testing of some important evolutionary questions that are difficult to monitor in open field populations, such as prediction of the nature of behavioural and physiological resistance strategies against specific interventions. Of particular relevance is the use of SFS studies to determine the feasibility of disease control strategies based on the release of sterile and/or genetically-modified refractory mosquitoes [72]. Despite the optimism that such novel control strategies have garnered [72], so far investigation of their feasibility has been largely based on laboratory studies [72]. It remains unknown whether mosquitoes carrying GM sterility or refractory traits would be fit enough to compete for female mates with their wild counterparts [72]. As unrestricted field trials of GM mosquitoes are unlikely to be authorized before all potential biosecurity risks have been evaluated [72], testing the viability of these mosquitoes under contained semi-field settings will be a mandatory first step before proceeding

## Conclusions

The present study reports the successful establishment of a self-propagating malaria vector population in an enclosed semi natural environment. Such populations provide a valuable new research tool for the experimental study of malaria vector ecology, evolution and control under environmental conditions that are largely representative of natural conditions. Climatic conditions within the SFS were broadly similar to those within natural *Anopheles *transmission settings and mosquitoes within the system exhibited similar demographic, life-history and behavioural traits to those in the field. This development will help facilitate the development and optimization of many vector control strategies, including the long-awaited transfer of new genetic control technologies from the laboratory to field application.

## Competing interests

The authors declare that they have no competing interests.

## Authors' contributions

KRN, BGJK and HMF designed this study. KRN and DM carried out the laboratory and SFS work. KRN analyzed and interpreted the data. KRN, HMF, BGJK drafted the manuscript. All authors read and approved the manuscript.
